# Measurement-level clustering of ligamentum flavum thickness identifies distinct phenotypes associated with lumbar lordosis in adult spinal deformity: a proof-of-concept study

**DOI:** 10.3389/fsurg.2026.1888647

**Published:** 2026-10-14

**Authors:** Dylan Glaser, Ahmad K. AlMekkawi, Ashlesha Bhojane, Ali Ebada, Christopher Carron, Salah G. Aoun, Andrew Abumoussa, Carlos A. Bagley

**Affiliations:** 1 School of Medicine, University of Missouri-Kansas City, Kansas City, MO, United States; 2 Department of Neurosurgery, Saint Luke’s Marion Bloch Neuroscience Institute, Kansas City, MO, United States; 3 Department of Neurosurgery, University of Texas Southwestern Medical Center, Dallas, TX, United States

**Keywords:** adult spinal deformity, clustering, ligamentum flavum, lumbar lordosis, machine learning, spinal stenosis

## Abstract

**Study design:**

Retrospective cohort study.

**Objectives:**

To identify distinct ligamentum flavum (LF) thickness phenotypes in adult spinal deformity (ASD) using measurement-level clustering and to characterize their association with traditional spinopelvic parameters in a proof-of-concept analysis.

**Methods:**

LF thickness was measured on preoperative MRI in 44 consecutive ASD patients (2018–2023), yielding 1,250 measurements; complete clinical and radiographic data were available in 40. K-means clustering (*k* = 3) was performed at the measurement level, treating thickness regimes as exploratory and unsupervised. Patients were assigned to a dominant phenotype, and all inferential comparisons were performed at the patient level. Between-phenotype differences in spinopelvic parameters were assessed with independent-samples *t*-tests and Mann–Whitney *U*-tests, with Hedges g effect sizes. Preliminary clinical and radiological outcomes were reported descriptively.

**Results:**

Three measurement-level thickness regimes were identified: Low LF (2.21 ± 0.51 mm, *n* = 619 measurements), Medium LF (3.92 ± 0.56 mm, *n* = 499), and High LF (6.11 ± 0.90 mm, *n* = 132). High-cluster measurements were distributed across 32 patients, but only one patient had High LF as a dominant phenotype and was excluded from comparisons (43 patients analyzed; 25 Low, 18 Medium). Lumbar lordosis differed significantly between Low and Medium phenotypes (Low: −44.6 ± 12.0° vs. Medium: −32.5 ± 8.9°; *p* = 0.003; Hedges g = −1.10, 95% CI −1.81 to −0.39), a large effect that was robust to nonparametric testing. Cluster purity was higher in Low LF (61.9% vs. 53.3%, *p* = 0.027; g = 0.70). Principal component analysis of standard parameters showed that mean LF thickness was moderately correlated with the dominant pelvic-morphology component (*r* = −0.44, *p* = 0.008), indicating that LF thickness is partially related to, rather than independent of, traditional parameters. Intra-rater reliability, assessed by blinded re-measurement of 15 patients, was moderate [ICC(2,1) = 0.632, 95% CI 0.242–0.855; Cohen kappa for phenotype = 0.524], and sensitivity analysis showed that the phenotype-lordosis association does not survive substitution of the re-read values (*p* = 0.005 to *p* = 0.245). In contrast, the continuous rank correlation does (rho =  + 0.496, *p* = 0.0015).

**Conclusions:**

Measurement-level clustering identifies distinct LF thickness phenotypes in ASD that are associated with lumbar lordosis. These exploratory findings establish a methodological foundation and motivate larger, outcome-based studies with formal measurement-reliability assessment to determine clinical utility.

## 1 Introduction

Adult spinal deformity (ASD) represents a significant clinical challenge affecting an estimated 32% of adults over age 60, with prevalence increasing alongside the aging global population (1–3). Surgical correction through long-segment posterior spinal fusion offers substantial improvement in pain, disability, and quality of life. However, these extensive procedures carry considerable risk, with overall complication rates ranging from 40% to 86% depending on patient factors and surgical complexity (4–6).

Among the most clinically significant complications following ASD surgery are proximal junctional kyphosis (PJK) and proximal junctional failure (PJF), occurring in 20%–40% and up to 35% of patients, respectively (7–10). These complications often necessitate revision surgery and are associated with increased morbidity, healthcare costs, and diminished patient satisfaction. Despite extensive research identifying risk factors, including age, bone mineral density, sagittal alignment, and surgical factors, predictive accuracy remains suboptimal (11–13).

The ligamentum flavum (LF) is a posterior spinal structure connecting adjacent vertebral laminae and contributing to spinal canal integrity (14–16). LF hypertrophy, characterized by increased thickness exceeding 4–5 mm, is a well-established cause of lumbar spinal stenosis and is associated with degenerative disc disease, facet joint arthropathy, and advancing age (17–20). Histopathological studies have demonstrated that hypertrophied LF exhibits decreased elastic fiber content, increased collagen deposition, and fibrosis (21–23). However, the implications of LF heterogeneity—variation in LF thickness across spinal levels within individual patients—remain unexplored in ASD.

Traditional spinopelvic measurements—including pelvic incidence (PI), lumbar lordosis (LL), pelvic tilt (PT), sacral slope (SS), thoracic kyphosis (TK), and sagittal vertical axis (SVA)—are well-established descriptors of spinal alignment in ASD (24–26). Whether imaging-derived LF thickness patterns relate to, or add information beyond, these established parameters has not been examined.

To address this gap, we applied measurement-level K-means clustering to identify LF thickness phenotypes in patients with ASD. This proof-of-concept study aims to: (1) establish that distinct, reproducible LF thickness regimes can be identified; (2) characterize the resulting patient-level phenotypes and their association with spinopelvic parameters; and (3) examine, in an exploratory manner, the relationship between LF thickness and traditional radiographic parameters. We frame this work explicitly as hypothesis-generating rather than as a validated prediction tool (36).

## 2 Materials and methods

### 2.1 Study design and patient selection

This retrospective cohort study was conducted at Saint Luke's Hospital of Kansas City with institutional review board approval. We included consecutive adult patients (≥18 years) who underwent surgical correction for ASD between January 2018 and December 2023. Inclusion criteria required a preoperative lumbar MRI of sufficient quality for LF measurement and a minimum 3-level posterior spinal fusion. Patients with prior lumbar surgery at measured levels, active spinal infection, or tumor-related deformity were excluded.

### 2.2 Cohort flow and analytic sets

LF thickness measurements were obtained in 44 patients (1,250 measurements), which constituted the cohort for measurement-level clustering and patient-level phenotype assignment. Complete clinical and radiographic data were available in 40 of these patients, who comprised the analytic cohort for clinical and radiographic comparisons; four patients had LF measurements without linked clinical records and were therefore included only in the clustering analysis. The number of patients contributing to each analysis is reported explicitly throughout the Results and in the table footnotes.

### 2.3 LF thickness measurement

LF thickness was measured on axial T2-weighted MRI sequences at each lumbar level (L1-L2 through L5-S1), following established protocols (17, 27). At each level, the LF was identified as the hypointense structure connecting adjacent laminae. Thickness was measured perpendicular to the ligament at its maximum extent, from the inner boundary at the dural surface to the outer boundary against the lamina; the full protocol, including the operative definition of each boundary, is illustrated in Figure 1 and set out step by step in Supplementary Table S1. Multiple measurements were obtained per level to capture within-level and within-patient heterogeneity. Specifically, thickness was recorded bilaterally at every axial slice spanning each level, with several measurements per slice, yielding approximately six measurements per level per patient (1,250 in total). Sampling the ligament bilaterally and across all slices rather than at a single midline point was intended to capture within-level asymmetry, including unilateral or asymmetric hypertrophy that a single measurement would miss. LF measurements were obtained without reference to the sagittal-alignment parameters or clinical outcomes.

**Figure 1:**
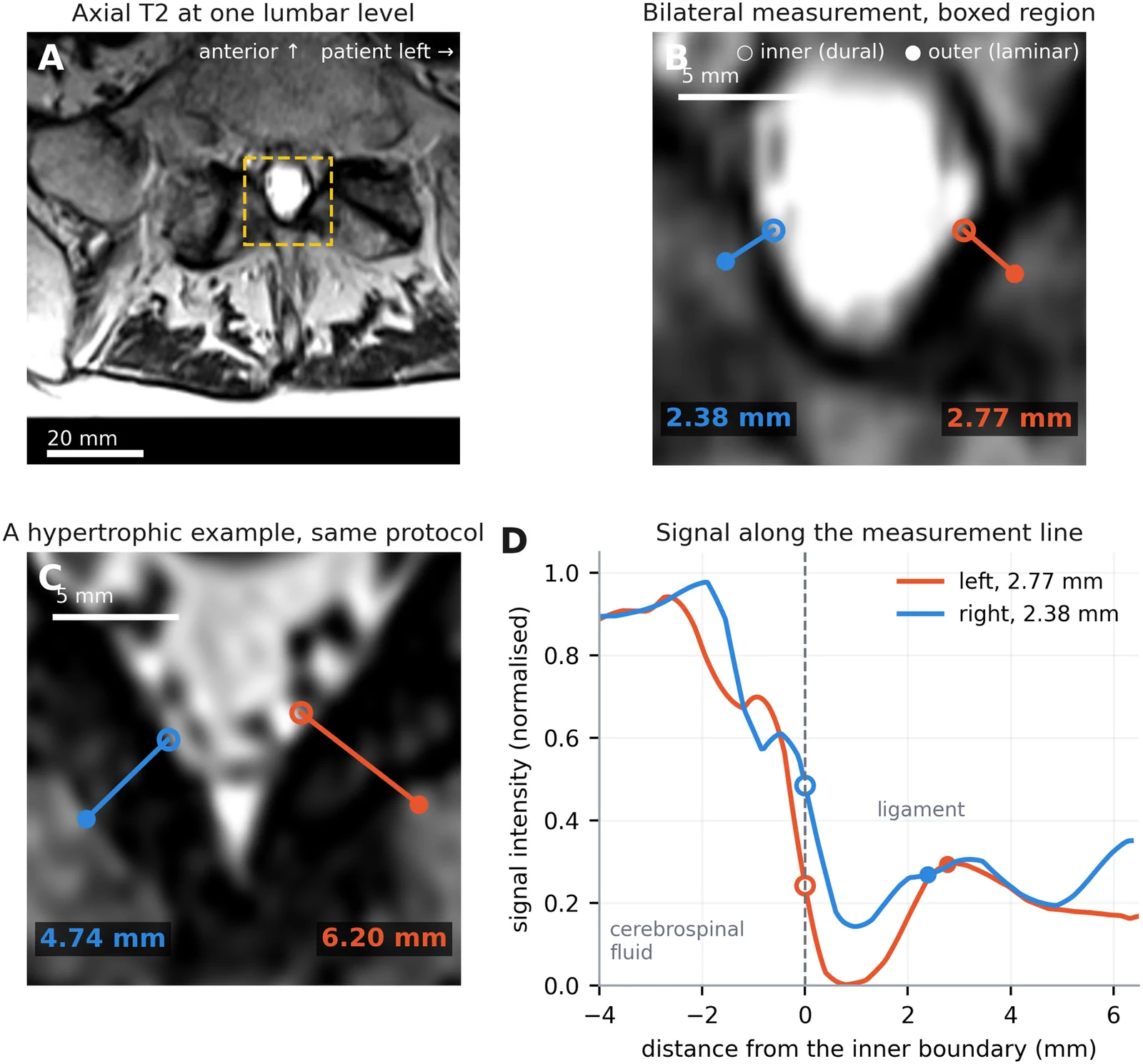
LF thickness measurement protocol. **(A)** Axial T2-weighted image at one lumbar level; anterior is at the top, and the patient is on the left, and the dashed box marks the measured region. **(B)** The boxed region enlarged, with the bilateral measurement shown: the open circle marks the inner boundary at the dural surface and the filled circle the outer boundary against the lamina, with thickness taken as the distance between them perpendicular to the ligament at its maximum extent. **(C)** A hypertrophic example measured under the identical protocol. **(D)** Signal intensity sampled along each measurement line, with the inner boundary at 0 mm; the transition out of cerebrospinal fluid defines the inner boundary, while the outer boundary abuts the lamina and is not marked by an equivalent intensity edge. Scale bars as shown.

All available slices were used, and no examinations were excluded for image quality. Acquisition was not homogeneous across the cohort. Parameters extracted from the DICOM headers of all 44 studies show six manufacturers (Siemens *n* = 23, GE *n* = 15, Hitachi *n* = 3, Philips *n* = 2, FONAR *n* = 1), field strengths from 0.3 T to 3.0 T (1.5 T *n* = 38, 3.0 T *n* = 3, 0.7 T *n* = 1, 0.6 T *n* = 1, 0.3 T *n* = 1), and in-plane resolution from 0.281 to 1.239 mm across 22 distinct values (Supplementary Table S2). Because acquisition varied, we tested whether it influenced the results: neither in-plane resolution nor field strength was associated with mean LF thickness (*r* =  + 0.000, *p* = 0.999 and *r* = −0.135, *p* = 0.38), neither differed between phenotypes (*p* = 0.93 and *p* = 0.21), and manufacturer was unrelated to phenotype (*p* = 0.96). Acquisition heterogeneity therefore does not appear to confound the reported associations, but it does limit measurement precision in individual studies, as addressed in the Limitations.

A single reader, a trained medical student, performed measurements. They were then independently reviewed and, where necessary, corrected by a spine research fellow, and finally verified by a fellowship-trained spine neurosurgeon. This three-tier sequential process is a consensus workflow rather than independent duplicate measurement: each value originates from one reader and is confirmed or adjusted at successive levels of expertise. It therefore does not by itself establish reliability, which we assessed separately as follows.

To quantify measurement repeatability, one reader independently re-measured a blinded, randomly ordered sample of 15 patients after a washout interval, following the same protocol and without access to the original values, the original slice positions, or any algorithmic estimate. Patients were selected to span the full cohort range of mean LF thickness (2.36–4.58 mm). Agreement was assessed with the intraclass correlation coefficient [ICC(2,1), two-way random effects, absolute agreement, single measure; 95% confidence interval by the method of McGraw and Wong], Bland-Altman analysis, the standard error of measurement (SEM), and the minimal detectable change (MDC95 = 1.96 × sqrt(2) × SEM). Concordance of the resulting dominant phenotype was assessed with Cohen kappa.

### 2.4 Measurement-level K-means clustering

Each LF measurement (*n* = 1,250) was treated as an observation for the purpose of identifying thickness regimes across the full measurement spectrum. We emphasize that this measurement-level step is an exploratory, unsupervised procedure for discretizing a continuous thickness distribution; it is not used for statistical inference. K-means clustering was performed with *k* = 3, with *k* selected using the elbow method and silhouette analysis (28, 29) and reported alongside a sensitivity analysis across *k* = 2 to *k* = 5 (Supplementary Table S3). Because clustering is performed on a single continuous variable, the procedure effectively identifies data-driven thickness cut points; we adopt the three-regime solution for clinical interpretability while noting that a two-regime solution yielded a marginally higher silhouette value.

### 2.5 Patient-level assignment and inference

Following measurement-level clustering, each patient was assigned to their dominant cluster—the cluster containing the majority of their measurements. Cluster purity was calculated as the proportion of measurements belonging to the dominant cluster, providing a patient-level summary of within-patient thickness homogeneity. All inferential comparisons were performed at the patient level (one observation per patient), thereby avoiding the non-independence inherent in treating multiple measurements per patient as independent observations. One patient whose dominant phenotype was High LF was excluded from between-group comparisons because a single patient cannot support inference; comparisons were therefore between Low LF and Medium LF phenotypes.

### 2.6 Relationship to traditional parameters

To examine whether LF thickness relates to traditional parameters, we performed principal component analysis (PCA) on eight standard baseline variables (age, BMI, PI, LL, PT, SS, TK, SVA) (33–35) in patients with complete data. Then we assessed the correlation between mean patient-level LF thickness and the resulting principal component scores. This approach avoids the circularity of including multiple collinear LF summary features within the PCA itself. PCA was treated as descriptive given the sample size, and the complete-case number is reported with the result.

### 2.7 Preliminary outcome assessment

To provide context for future outcome-based studies, available clinical and radiological follow-up data (ODI, VAS scores, and radiographic PJF status at 3, 6, and 12 months) were summarized descriptively by phenotype. Given the sample size and low event rate, these data are presented without inferential comparison, and no association with outcomes is claimed.

### 2.8 Statistical analysis

Patient-level continuous variables were compared using independent-samples *t*-tests with corresponding Mann–Whitney *U*-tests, reported as mean ± SD; effect sizes are reported as Hedges g. Categorical variables were compared using Fisher's exact test. Given the proof-of-concept nature and exploratory intent, no adjustment for multiple comparisons was applied, and all *p*-values are interpreted as nominal and hypothesis-generating. Statistical significance was set at *p* < 0.05. Analyses were performed in Python 3.9 using scikit-learn and scipy.

Two sensitivity analyses were performed to test whether the principal finding is robust to measurement error. First, the dominant phenotypes derived from the blinded re-read were substituted for the original assignments in the 15 re-measured patients, leaving all other patients unchanged, and the lordosis comparison was repeated. Second, replication was simulated by generating 4,000 re-measurements of the full cohort under a bivariate normal model in which a repeat measurement correlates with the original at the measured reliability (ICC 0.632) and reproduces the observed marginal variance. The proportion of simulated replications reaching *p* < 0.05 was recorded.

## 3 Results

### 3.1 Patient characteristics

LF measurements were obtained in 44 patients, yielding 1,250 individual measurements (mean 28.4 ± 6.2 per patient). Among the 40 patients with complete clinical data, the cohort was predominantly female (70.0%), with a mean age of 69.4 ± 9.5 years and a BMI of 27.3 ± 5.4 kg/m^2^. Baseline spinopelvic parameters (available in 35 patients) were: PI 56.3 ± 13.8°, LL −39.0 ± 12.1°, PT 28.1 ± 11.7°, SS 28.2 ± 8.2°, TK 26.0 ± 15.0°, and SVA 44.0 ± 52.8 mm (Table 1).

**Table 1:** Patient demographics and baseline characteristics (complete-case cohort, *n* = 40).

**Variable**	**Value**
Age, years	69.4 ± 9.5
Female, *n* (%)	28 (70.0%)
BMI, kg/m^2^	27.3 ± 5.4
PI,°	56.3 ± 13.8
LL,°	−39.0 ± 12.1
PT,°	28.1 ± 11.7
SS,°	28.2 ± 8.2
TK,°	26.0 ± 15.0
SVA, mm	44.0 ± 52.8
LF measurements per patient	28.4 ± 6.2

Values are mean ± SD or *n* (%). Spinopelvic parameters available in 35 patients. PI, pelvic incidence; LL, lumbar lordosis; PT, pelvic tilt; SS, sacral slope; TK, thoracic kyphosis; SVA, sagittal vertical axis; LF, ligamentum flavum.

### 3.2 Identification of LF thickness regimes

Measurement-level K-means clustering (*k* = 3) identified three thickness regimes with good separation (silhouette score = 0.573). The Low LF cluster contained 619 measurements (49.5%, 2.21 ± 0.51 mm), the Medium LF cluster 499 measurements (39.9%, 3.92 ± 0.56 mm), and the High LF cluster 132 measurements (10.6%, 6.11 ± 0.90 mm). All pairwise differences in thickness between clusters were, as expected for a thickness-based partition, highly significant (Table 2, Figure 2). A sensitivity analysis across *k* = 2 to *k* = 5 is provided in Supplementary Table S3; a two-regime solution yielded a marginally higher silhouette value (0.598), and the three-regime solution was retained for interpretability.

**Table 2:** LF thickness regimes at the measurement level (*n* = 1,250 measurements).

**Regime**	***n* (%)**	**Mean ± SD (mm)**	**Range (mm)**	**Patients (dominant)**
Low LF	619 (49.5%)	2.21 ± 0.51	0.7–3.0	25
Medium LF	499 (39.9%)	3.92 ± 0.56	3.1–5.0	18
High LF	132 (10.6%)	6.11 ± 0.90	5.1–9.5	1

High-cluster measurements were distributed across 32 patients; only one patient had High LF as the dominant phenotype. Silhouette score (*k* = 3) = 0.573. Thickness differences between regimes are inherent to the partition.

**Figure 2:**
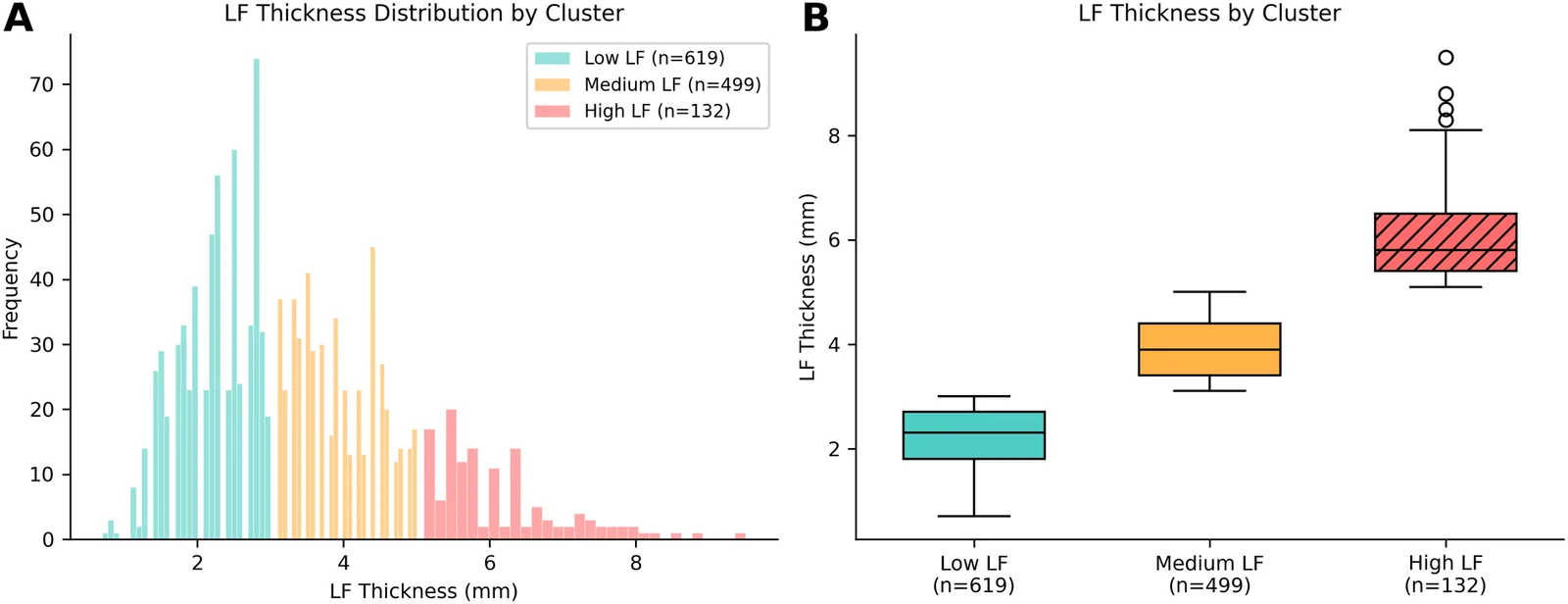
LF thickness distribution by cluster. **(A)** Overlapping histograms showing measurement distribution within each thickness regime. **(B)** Box plots demonstrating separation between regimes. The High LF regime is shown with a hatched pattern.

### 3.3 Patient-level phenotypes and within-patient heterogeneity

At the patient level, 25 patients (56.8%) had a Low LF dominant phenotype, 18 (40.9%) a Medium LF phenotype, and 1 (2.3%) a High LF phenotype. Notably, the 132 High-cluster measurements were distributed across 32 different patients rather than concentrated in a single individual, indicating that thick-LF measurements occur sporadically across the cohort; only one patient (a 77-year-old; mean LF 4.25 mm; cluster purity 35.7%) had High LF as the dominant pattern. This single patient was excluded from between-group comparisons, leaving 43 patients for patient-level analysis (Figure 3).

**Figure 3:**
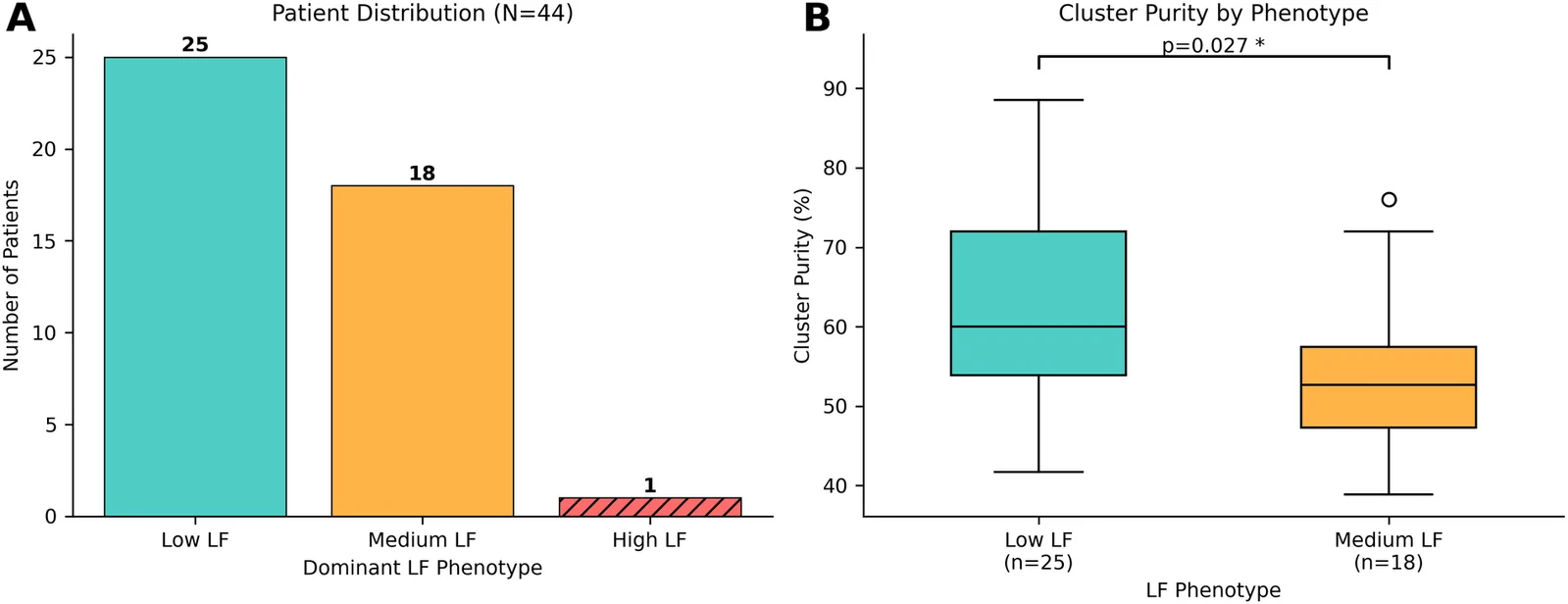
Patient-level phenotype results. **(A)** Distribution of patients across dominant LF phenotypes: Low LF (*n* = 25), Medium LF (*n* = 18), High LF (*n* = 1). **(B)** Cluster purity comparison between Low LF (61.9 ± 13.6%) and Medium LF (53.3 ± 9.7%) phenotypes (*p* = 0.027). *p < 0.05.

### 3.4 Association of phenotype with lumbar lordosis

Among patients with complete radiographic data, lumbar lordosis differed significantly between phenotypes, with the Low LF group demonstrating greater lordosis (Low: −44.6 ± 12.0°, *n* = 19 vs. Medium: −32.5 ± 8.9°, *n* = 15; *p* = 0.003, Mann–Whitney *p* = 0.003; Hedges g = −1.10, 95% CI −1.81 to −0.39). This represents a large effect that was robust to nonparametric testing. Smaller, non-significant differences favoring the Low LF group were observed for PI (*p* = 0.078; g = 0.61) and SS (*p* = 0.075; g = 0.62). No significant differences were found for age, BMI, sex, PT, TK, or SVA (all *p* > 0.17) (Table 3, Figure 4). Mean LF thickness differed between groups by construction (Low: 2.95 ± 0.44 mm vs. Medium: 3.77 ± 0.47 mm; *p* < 0.001; g = −1.78).

**Table 3:** Patient-Level comparison of Low LF vs. Medium LF Phenotypes.

**Variable**	**Low LF**	**Medium LF**	***p* (t/MW)**	**Hedges g**
Age, years	68.7 ± 10.0 (23)	69.8 ± 9.0 (16)	0.73/0.57	−0.11
Female, *n* (%)	16 (69.6%)	12 (75.0%)	0.99 (Fisher)	–
BMI, kg/m^2^	28.1 ± 5.7 (23)	25.7 ± 4.5 (16)	0.18/0.21	+0.44
Mean LF, mm	2.95 ± 0.44 (25)	3.77 ± 0.47 (18)	<0.001/<0.001	−1.78
Cluster purity, %	61.9 ± 13.6 (25)	53.3 ± 9.7 (18)	0.027/0.023	+0.70
PI,°	60.2 ± 13.3 (19)	51.8 ± 13.7 (15)	0.078/0.089	+0.61
LL,°	−44.6 ± 12.0 (19)	−32.5 ± 8.9 (15)	0.003/0.003	−1.10
PT,°	29.7 ± 12.4 (19)	26.3 ± 11.3 (15)	0.42/0.40	+0.27
SS,°	30.6 ± 7.6 (19)	25.5 ± 8.6 (15)	0.075/0.115	+0.62
TK,°	27.7 ± 15.8 (19)	23.5 ± 14.5 (15)	0.44/0.42	+0.26
SVA, mm	36.7 ± 47.6 (19)	51.0 ± 60.5 (15)	0.45/0.43	−0.26

Values are mean ± SD (*n*) or *n* (%). The single High LF patient was excluded. *p*-values from independent-samples *t*-test (*t*) and Mann–Whitney *U*-test (MW); all nominal and unadjusted for multiple comparisons. Mean LF differs by construction. Bold significance threshold *p* < 0.05. 95% confidence intervals for Hedges g (normal approximation): Age −0.11 (−0.73, +0.52); BMI +0.44 (−0.20, +1.07); mean LF −1.78 (−2.48, −1.07); cluster purity +0.70 (+0.08, +1.31); PI +0.61 (−0.06, +1.29); LL −1.10 (−1.81, −0.39); PT +0.27 (−0.39, +0.94); SS +0.62 (−0.06, +1.30); TK +0.26 (−0.40, +0.93); SVA −0.26 (−0.92, +0.40).

**Figure 4:**
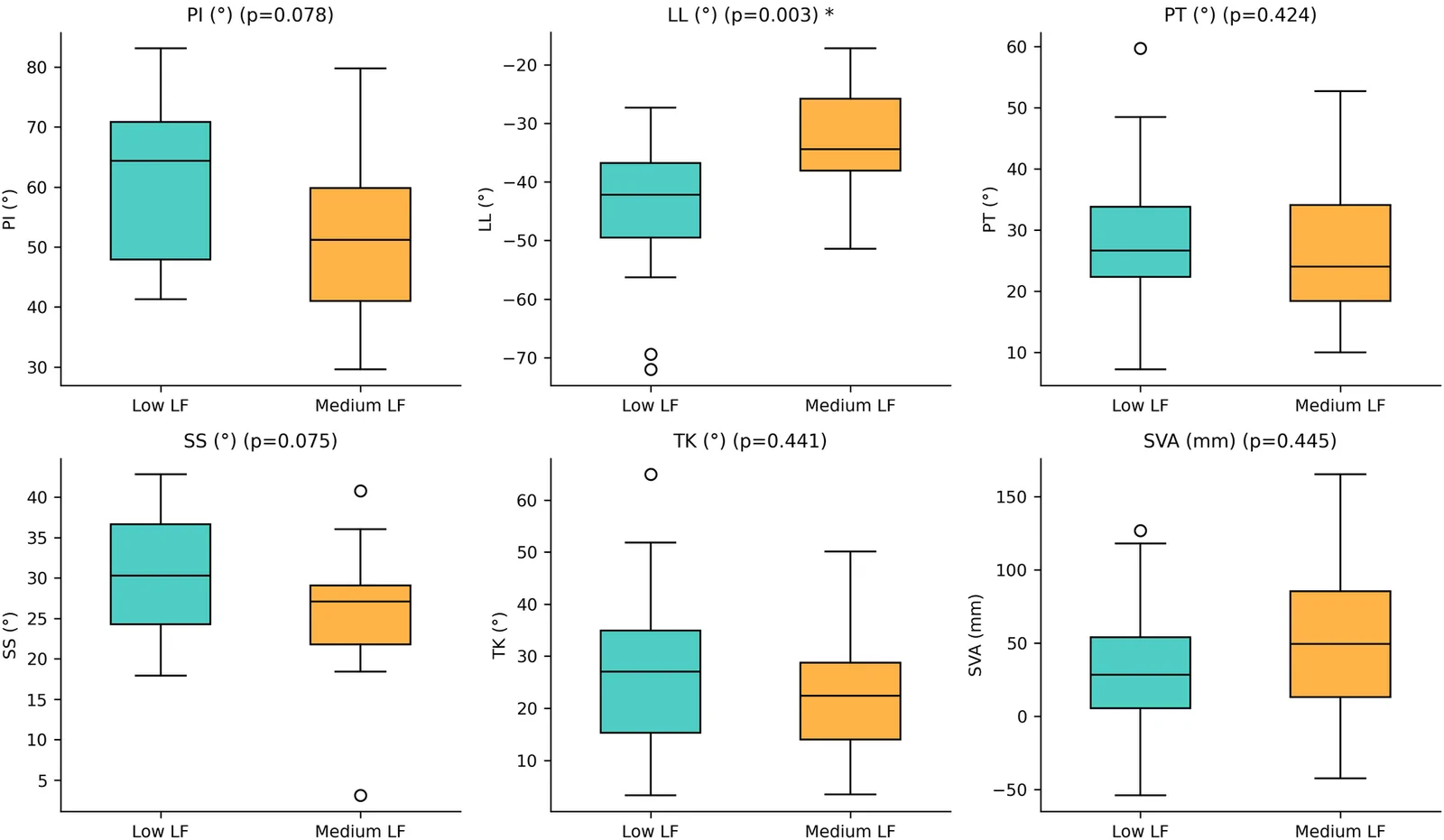
Radiographic parameters by LF phenotype (complete-case data). Six-panel comparison of PI, LL, PT, SS, TK, and SVA. A significant, large-effect difference was observed for lumbar lordosis (*p* = 0.003). *p < 0.05.

### 3.5 Cluster purity

Cluster purity was higher in Low LF patients (61.9 ± 13.6%) than Medium LF patients (53.3 ± 9.7%; *p* = 0.027, Mann–Whitney *p* = 0.023; Hedges g = 0.70), indicating that patients with predominantly thinner LF tended to have more homogeneous thickness across levels (Figure 3B).

### 3.6 Measurement reliability

Intra-rater agreement for mean LF thickness was moderate: ICC(2,1) = 0.632 (95% CI 0.242–0.855; *n* = 15 patients). The re-read was systematically thicker than the original by 0.357 mm, with 95% limits of agreement of −0.81 to +1.53 mm, SEM 0.465 mm and MDC95 1.289 mm (Supplementary Figures S1A,B). Reliability was level-dependent, ranging from ICC 0.222 at L1-L2 to 0.786 at L5-S1 (Supplementary Figure S1C, Table S4). Concordance of the dominant phenotype was moderate (Cohen kappa = 0.524): 4 of 15 patients (27%) changed phenotype between readings, in every case between the Low and Medium regimes (Supplementary Figure S1, Table S5).

An MDC95 of 1.289 mm means that a difference smaller than this, between two measurements of the same patient, cannot be distinguished from measurement error. This applies to individual patients and not to comparisons of group means, which are estimated far more precisely. It does, however, bear directly on phenotype assignment, because the Low-Medium cluster boundary lies 1.71 mm from either center.

Both prespecified sensitivity analyses are reported here. Substituting the re-read phenotypes for the 15 re-measured patients changed the assignment of 4 patients and moved the phenotype-lordosis comparison from *p* = 0.005 to *p* = 0.245 (Hedges g −0.96 to −0.39). In the simulated replication, the categorical comparison reached *p* < 0.05 in 6% of 4,000 replications, while the continuous rank correlation between mean LF thickness and lumbar lordosis reached *p* < 0.05 in 29%. The implications are addressed in the Limitations.

### 3.7 Relationship between LF thickness and traditional parameters

PCA of the eight standard baseline parameters (complete-case *n* = 35) yielded a first component driven primarily by pelvic morphology (PI, BMI, SS loadings highest) and a second component driven by sagittal alignment (LL, SVA, PT). Mean patient-level LF thickness was moderately and significantly correlated with the first (pelvic-morphology) component (*r* = −0.44, *p* = 0.008) and was not significantly correlated with the remaining components. Rather than being independent of traditional parameters, LF thickness therefore appears partially related to spinopelvic morphology, consistent with its observed association with lumbar lordosis (Figure 5). Given the small complete-case sample relative to the number of variables, this analysis is descriptive and should be interpreted with caution.

**Figure 5:**
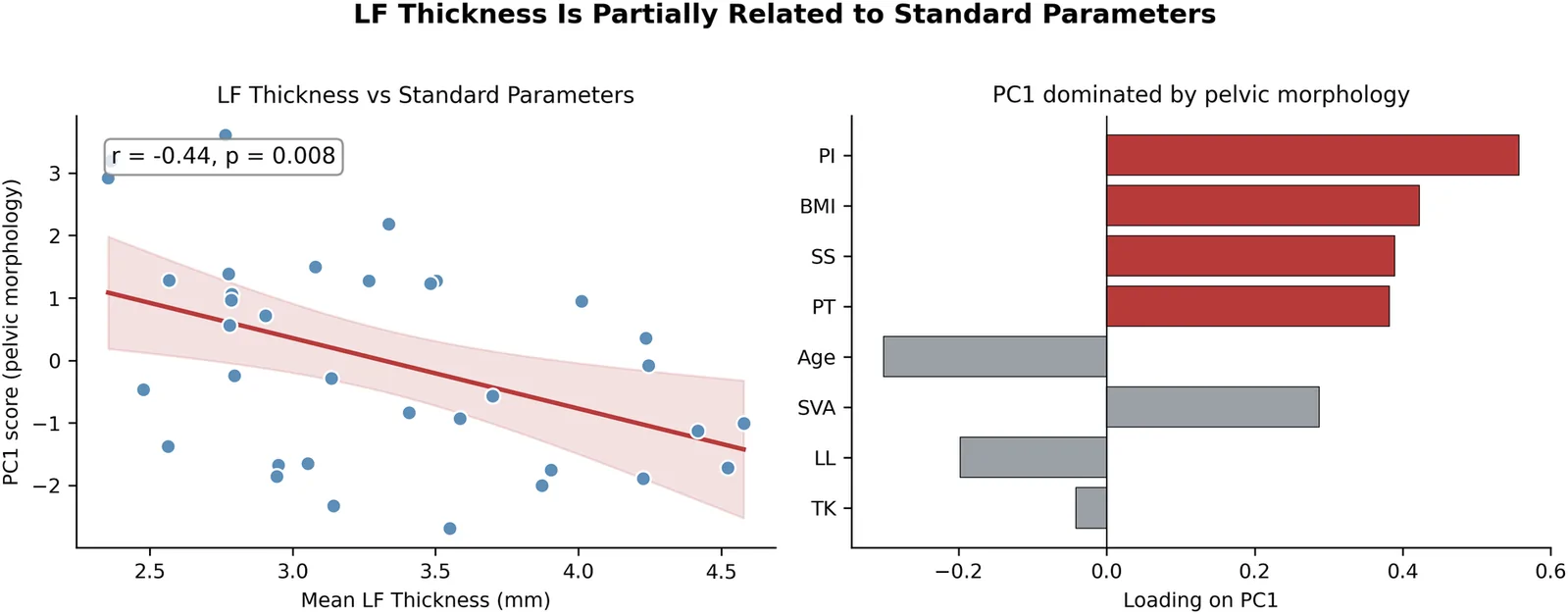
Relationship between LF thickness and traditional parameters. Principal component analysis of eight standard baseline parameters, with mean patient-level LF thickness correlated against component scores. Mean LF thickness was moderately correlated with the pelvic-morphology component (*r* = −0.44, *p* = 0.008), indicating partial relationship rather than independence.

Examined directly as a continuous variable rather than as a discretized phenotype, mean LF thickness correlated with lumbar lordosis (Spearman rho =  + 0.496, *p* = 0.0015; Pearson *r* =  + 0.388, *p* = 0.016; *n* = 38). Because lumbar lordosis is expressed as a negative value, a positive correlation indicates that greater LF thickness accompanies less lordosis, the same direction as the phenotype comparison. This continuous form of the association also survived substitution of the re-read values (rho =  + 0.417, *p* = 0.0091).

### 3.8 Preliminary clinical outcomes

Available follow-up data are summarized descriptively in Table 4. A single PJF event was observed at 3 months (in the Low LF group), with no further events at 6 or 12 months, and follow-up completeness varied across timepoints. Given the single event and variable follow-up, no association between phenotype and PJF, revision, screw loosening, or patient-reported outcomes can be inferred; these data are presented solely to establish feasibility for larger studies.

**Table 4:** Descriptive clinical and radiological outcomes by LF phenotype.

**Variable**	**Low LF**	**Medium LF**
ODI Baseline	37.6 ± 10.5 (21)	34.6 ± 12.3 (14)
ODI 6 months	30.9 ± 11.6 (13)	28.4 ± 10.3 (10)
ODI 12 months	31.3 ± 12.6 (12)	36.3 ± 13.3 (7)
VAS Back Baseline	5.6 ± 2.4 (22)	6.9 ± 2.8 (14)
VAS Back 12 months	4.9 ± 2.5 (13)	4.2 ± 1.8 (10)
VAS Leg Baseline	4.6 ± 3.1 (22)	4.0 ± 3.8 (14)
VAS Leg 12 months	3.1 ± 2.9 (13)	1.4 ± 2.2 (10)
PJF 3 months, n/N (%)	1/21 (4.8%)	0/15 (0.0%)
PJF 6 months, n/N (%)	0/16 (0.0%)	0/9 (0.0%)
PJF 12 months, n/N (%)	0/10 (0.0%)	0/6 (0.0%)

Values are mean ± SD (*n* with available data) or n/N (%). Data are descriptive; sample sizes and event rates are insufficient for inferential comparison, and no association with outcomes is claimed. ODI, Oswestry Disability Index; VAS, visual analog scale; PJF, proximal junctional failure.

## 4 Discussion

This proof-of-concept study applied measurement-level clustering to characterize heterogeneity in ligamentum flavum thickness in adult spinal deformity. Three findings emerged: (1) reproducible LF thickness regimes can be identified and used to assign patient-level phenotypes; (2) these phenotypes are associated with lumbar lordosis, with a large effect size; and (3) LF thickness is partially related to, rather than independent of, traditional spinopelvic parameters. We interpret these findings as hypothesis-generating and as motivation for larger, outcome-based investigation.

### 4.1 Methodological considerations

Traditional analyses of LF thickness aggregate measurements at the patient level, obscuring variation across spinal levels (17, 27). Abbas et al. demonstrated that LF thickness varies substantially across lumbar levels, with L4-L5 typically showing the greatest hypertrophy (17). In the present ASD cohort, however, LF thickness was relatively uniform across L1–S1 (level means 3.06–3.46 mm), and the thickness regimes were distributed across all lumbar levels rather than concentrated at L4-L5: the proportion of High-regime measurements ranged from 8.8% (L5-S1) to 11.9% (L3-L4), with L4-L5 at 10.9% (Supplementary Table S5). The strata therefore do not simply recapitulate spinal level, and pooling levels is unlikely to have driven the observed association. By first identifying thickness regimes at the measurement level and then assigning patients by dominant pattern, our approach retains a summary of within-patient heterogeneity through the cluster purity metric while keeping all inference at the patient level. Because clustering was performed on a single continuous variable, it is most accurately understood as data-driven discretization of a thickness distribution rather than as discovery of latent multivariate structure; the marginally higher silhouette value for a two-regime solution is consistent with this interpretation. The value of the approach is therefore not that it uncovers hidden phenotypes, but that the resulting strata show a consistent and clinically interpretable association with sagittal alignment.

### 4.2 Association with lumbar lordosis

The principal finding is a significant association between LF phenotype and lumbar lordosis (*p* = 0.003; Hedges g = −1.10), with the thinner-LF phenotype demonstrating greater lordosis. This is consistent with biomechanical models proposing that mechanical stress contributes to LF hypertrophy through tissue microtrauma, inflammation, and fibrosis (20): reduced lordosis may increase compressive and shear loading on the posterior elements, plausibly promoting hypertrophic change. The smaller, same-direction trends for PI and SS, which are interrelated pelvic-morphology parameters (24), are consistent with this interpretation but did not reach significance in this small sample. We note that this is an association in cross-sectional data and does not establish causation or directionality.

### 4.3 Relationship to traditional parameters

An earlier framing of this work suggested that LF thickness captured information orthogonal to traditional parameters. On reanalysis using a single, non-redundant LF summary, this is not supported: mean LF thickness correlates moderately with the dominant pelvic-morphology component (*r* = −0.44, *p* = 0.008). The more defensible conclusion is that LF thickness is partially related to spinopelvic morphology rather than independent of it—a conclusion that is, in fact, more consistent with the observed lordosis association. We therefore make no claim that LF phenotyping provides information independent of, or additive to, standard radiographic assessment; establishing incremental value would require a dedicated, adequately powered analysis. The original 21-variable principal component analysis, retained from the first submission for completeness, is provided as Supplementary Tables S6 to S8 and Supplementary Methods; it is superseded by the reanalysis reported here.

### 4.4 Within-patient heterogeneity

The distribution of high-thickness measurements across 32 patients—despite only one patient having a High LF dominant phenotype—illustrates that focal thick-LF measurements are common even in patients whose overall pattern is not hypertrophic. Cluster purity, which differed between phenotypes (*p* = 0.027; g = 0.70), provides a simple quantification of this heterogeneity. Park et al. demonstrated that focal LF hypertrophy is associated with localized inflammatory changes (19); whether cluster purity carries independent prognostic meaning is an open question for future work and is not addressed by the present data.

### 4.5 Limitations

Second, and most importantly for a measurement-derived phenotype, measurement reliability constrains what can be inferred. Intra-rater agreement was moderate (ICC 0.632, 95% CI 0.242–0.855) with a systematic bias of +0.357 mm, and 27% of re-measured patients changed dominant phenotype (kappa = 0.524). Because the Low-Medium cluster boundary lies 1.71 mm from either cluster center and the MDC95 is 1.289 mm, phenotype assignment for an individual patient is not reliable at the achieved precision. We tested the consequence directly rather than leaving it to inference: substituting the re-read phenotypes for 15 patients moved the phenotype-lordosis comparison from *p* = 0.005 to *p* = 0.245, and only 6% of simulated replications reproduced a significant categorical result. The magnitude of the association reported here is therefore likely to be inflated, and this cohort at this measurement reliability cannot establish its size with confidence. The direction of the association and the continuous rank correlation (rho =  + 0.496, surviving substitution at *p* = 0.009, and significant in 29% of simulated replications) are the more durable findings. This reinforces the hypothesis-generating framing of the study; it does not rescue the categorical result, and we do not present it as established.

Third, acquisition heterogeneity limits measurement precision in individual studies. In-plane resolution varied 4.4-fold across the cohort; at the coarsest resolution (1.239 mm), a 3 mm ligament spans fewer than three pixels, and quantization alone contributes approximately +/−0.6 mm. The single patient with a High LF dominant phenotype was imaged on a 0.6T open-configuration system at this coarse resolution, and that phenotype assignment should be regarded as provisional.

Fourth, LF thickness measurement is sensitive to conditions that are not usually recorded. In a controlled experiment on a subset of these images, the same reader measuring identical images produced session mean values of 2.20, 1.48 and 2.84 mm across three sittings [ICC(2,1) = 0.264], and varying the display window across four settings changed the mean reading systematically [F(3,33) = 9.93, *p* < 0.001], with a range of 2.44 mm across windows for the same slice and side (Supplementary Figure S2). Measurements in this study were made in a single reading session at one display setting that was not recorded. We report this as a limitation on comparability rather than as a finding about the wider literature; the experiment was conducted in a purpose-built annotation environment rather than in the clinical viewer used for the study measurements, and its magnitude should not be assumed to transfer directly. It does, however, indicate that display settings and reading-session structure should be specified in protocols for quantitative LF measurement.

Fifth, clustering was performed on a single continuous variable and is sensitive to the choice of k; we report this transparently. Sixth, measurements pooled lumbar levels (L1-S1) without level-specific normalization, and level-stratified analysis is a natural extension; the level-dependent reliability reported in Section 3.6 strengthens the case for it. In addition, because thickness was measured on standard axial slices rather than on images reformatted perpendicular to the canal at each level, coronal deformity or axial rotation may introduce oblique-plane measurement error at individual slices; this error is expected to be non-differential with respect to phenotype and is mitigated by averaging many measurements per patient, but orthogonal multiplanar reformatting is a refinement for future multi-center work. Seventh, the clinical rationale links lumbar LF thickness to proximal junctional pathology only indirectly; if LF thickness reflects generalized posterior soft-tissue degeneration, this must be tested directly rather than assumed, particularly because the proximal junction often lies above the measured lumbar levels. Eighth, this study does not evaluate clinical outcomes: with a single PJF event and variable follow-up, no outcome association can be inferred. Finally, no histopathological correlation was performed, so biological interpretations remain speculative.

### 4.6 Future directions

The natural next step is a multi-institutional study with formal measurement-reliability assessment, level-specific analysis, and sufficient enrollment and follow-up to test whether LF phenotype or cluster purity relates to clinically meaningful endpoints including PJK/PJF incidence, minimum clinically important difference achievement, and revision surgery. A target enrollment of several hundred patients across multiple centers would be required to populate the High LF phenotype and to support outcome modeling.

Automated LF measurement is the practical prerequisite for a study of that size, and it is also what would be required for LF thickness to become usable in surgical planning rather than in research alone (30). Contemporary planning frameworks in ASD are built on sagittal alignment measured from standing radiographs and on pelvic morphology (24, 25, 31, 32); they contain no measure of the posterior soft-tissue column. The ligamentum flavum is the most practical candidate for such a measure, being quantifiable on preoperative MRI that these patients already undergo. The present study indicates why it is not usable for that purpose today. The reason is measurement rather than biology: characterizing the ligament properly required 1,250 measurements across 44 patients, which is not achievable in clinical time, and at the achieved reliability an individual patient's phenotype is not stable between readings (MDC95 1.289 mm against a cluster boundary 1.71 mm from either center; 27% reassignment). Together, these findings are, in our view, a specific argument for automated segmentation as the necessary route to a posterior soft-tissue planning variable rather than merely a convenient one.

Three components that such a method requires are supplied here. An operative definition of the measurement target, including the observation that the inner boundary corresponds to a signal transition while the outer boundary abuts the lamina and is not marked by an equivalent edge, is given in Figure 1 and Supplementary Table S1 in implementable detail. A validation benchmark is provided by the reliability results in Section 3.6, which define the agreement an automated method would need to match a human reader and equally identify accuracy claims that would exceed what the reference standard itself supports. And the target quantity is specified: a segmentation mask is not a planning input until the summary computed from it is defined, and the within-patient heterogeneity documented here, with high-thickness measurements occurring in 32 of 44 patients while only one shows a predominantly hypertrophic pattern, indicates that a per-level thickness map rather than a single patient-level number is what should be produced. We emphasize that this study demonstrates neither clinical utility nor a relationship to any outcome; whether posterior soft-tissue state adds anything to existing alignment-based frameworks is precisely the question that automated measurement would make it possible to test.

### 4.7 Conclusion

Measurement-level clustering identifies distinct LF thickness phenotypes in adult spinal deformity that are associated with lumbar lordosis, with a large effect size. LF thickness appears partially related to spinopelvic morphology rather than independent of it. These exploratory findings establish a methodological foundation and motivate larger, outcome-based studies—incorporating formal measurement-reliability assessment—to determine whether LF phenotyping has clinical utility in ASD.

## Data Availability

The raw data supporting the conclusions of this article will be made available by the authors, without undue reservation.

## Supplementary material

Supplementary Table S1 Standard operating procedure for LF thickness measurement.

Supplementary Table S2 Acquisition parameters across the 44 studies.

Supplementary Table S3 Silhouette sweep for k = 2 to 5.

Supplementary Table S4 Intra-rater reliability by lumbar level.

Supplementary Table S5 Distribution of thickness regimes by lumbar level.

Supplementary Tables S6-S8 and Supplementary Methods Carried forward from the previous submission.

Supplementary Figure S1 Intra-rater reliability.

Supplementary Figure S2 Sensitivity of the measurement to reading conditions.
