# Supplementary material for: Measurement-level clustering of ligamentum flavum thickness identifies distinct phenotypes associated with lumbar lordosis in adult spinal deformity: a proof-of-concept study
Source: Front Surg. 2026 Oct 14;13:1888647. doi: 10.3389/fsurg.2026.1888647 (PMC13713654; doi:10.3389/fsurg.2026.1888647)
Supplement: Supplementary file 1 [file Table1.docx]

**Supplementary Figure Legends**

Supplementary Figure S1. Intra-rater reliability. (A) Original reading versus blinded re-read for patient-level mean LF thickness, with the line of identity. (B) Bland–Altman plot showing the systematic bias and 95% limits of agreement. (C) ICC(2,1) by lumbar level, with the pooled value shown as a dashed line.

Supplementary Figure S2. Sensitivity of the measurement to reading conditions. (A) Mean LF thickness measured by the same reader on identical images in three separate sessions. (B) Mean LF thickness by display window width, across four fixed settings. (C) Estimated contribution of each source of variation to a single reading, expressed as a standard deviation; pure noise is estimated from repeated presentations of the same image at the same window. Error bars are standard errors.
