# Supplementary material for: Measurement-level clustering of ligamentum flavum thickness identifies distinct phenotypes associated with lumbar lordosis in adult spinal deformity: a proof-of-concept study
Source: Front Surg. 2026 Oct 14;13:1888647. doi: 10.3389/fsurg.2026.1888647 (PMC13713654; doi:10.3389/fsurg.2026.1888647)
Supplement: Supplementary file 2 [file Table10.docx]

**Supplementary Table S8. Complete 21-variable cluster characteristics.**

| **Variable** | **High LF (n=16)** | **Sag Imbalance (n=6)** | **Typical ASD (n=22)** | **p** |
| --- | --- | --- | --- | --- |
| LF mean (mm) | 3.99 ± 0.34 | 2.81 ± 0.42 | 2.97 ± 0.32 | <0.001 |
| LF max (mm) | 7.24 ± 1.09 | 5.05 ± 0.69 | 5.50 ± 0.91 | <0.001 |
| Age (y) | 70.1 ± 9.9 | 60.7 ± 10.7 | 71.2 ± 6.6 | 0.033 |
| BMI | 26.6 ± 5.0 | 32.8 ± 7.2 | 26.3 ± 3.7 | 0.016 |
| PI (°) | 49.4 ± 9.1 | 70.6 ± 8.2 | 57.4 ± 11.6 | <0.001 |
| SS (°) | 24.2 ± 6.4 | 36.0 ± 4.6 | 29.0 ± 6.7 | 0.001 |
| SVA (mm) | 37.5 ± 46.6 | 109.4 ± 49.2 | 30.8 ± 30.7 | <0.001 |
| TPA (°) | -3.8 ± 4.5 | 2.9 ± 4.8 | -4.6 ± 3.4 | <0.001 |
| ODI | 32.9 ± 7.9 | 43.0 ± 11.1 | 36.9 ± 10.4 | 0.099 |
| VAS leg | 3.1 ± 3.1 | 4.3 ± 4.4 | 5.1 ± 2.4 | 0.145 |
| OR time (min) | 306 ± 76 | 388 ± 86 | 258 ± 70 | 0.002 |

*From the original 21-variable analysis; superseded by the reanalysis in Section 3.7.*
