# Supplementary material for: Measurement-level clustering of ligamentum flavum thickness identifies distinct phenotypes associated with lumbar lordosis in adult spinal deformity: a proof-of-concept study
Source: Front Surg. 2026 Oct 14;13:1888647. doi: 10.3389/fsurg.2026.1888647 (PMC13713654; doi:10.3389/fsurg.2026.1888647)
Supplement: Supplementary file 3 [file Table2.docx]

**Supplementary Methods**

*Carried forward from the previous submission. Section S4 describes the original 21-variable principal component analysis, which is superseded by the reanalysis reported in Sections 2.6 and 4.3 of the manuscript and is retained here for completeness.*

**S1. Software**

Python 3.9.7 with scikit-learn 1.0.2, pandas 1.4.2, numpy 1.22.3, matplotlib 3.5.1, seaborn 0.11.2, scipy 1.8.0.

**S2. K-Means Parameters**

Measurement-level: k=3, k-means++ initialization, random_state=42, n_init=10. Silhouette=0.573. Patient-level (21 variables): Same parameters on standardized data.

**S3. Outlier Exclusion**

Patient distribution: Low LF n=24 (54.5%), Medium LF n=19 (43.2%), High LF n=1 (2.3%). Single High LF patient excluded from between-group comparisons (n=1 precludes inference).

**S4. PCA Methodology**

21 variables across 5 domains: LF (6): mean, SD, min, max, 25th, 75th; Demographics (3): age, BMI, weight; Radiological (7): PI, LL, PT, SS, TK, TPA, SVA; Clinical (3): ODI, VAS back, VAS leg; Surgical (2): OR time, LOS. Z-score standardization. Missing values imputed with means.

**S5. TRIPOD+AI Compliance**

This study follows TRIPOD+AI guidelines: ML methodology identified in title; single-center retrospective design; clear inclusion/exclusion with outlier documentation; algorithm parameters fully described; silhouette and variance metrics reported; code available upon request.
