# Supplementary material for: Measurement-level clustering of ligamentum flavum thickness identifies distinct phenotypes associated with lumbar lordosis in adult spinal deformity: a proof-of-concept study
Source: Front Surg. 2026 Oct 14;13:1888647. doi: 10.3389/fsurg.2026.1888647 (PMC13713654; doi:10.3389/fsurg.2026.1888647)
Supplement: Supplementary file 4 [file Table3.docx]

**Supplementary Table S1. Standard operating procedure for LF thickness measurement.**

| **Step** | **Procedure** |
| --- | --- |
| 1. Sequence | Axial T2-weighted sequence covering L1-L2 through L5-S1. Use the acquisition as reconstructed; do not reformat. |
| 2. Level identification | Identify each disc level on the sagittal localiser and work through every axial slice spanning that level. |
| 3. Structure | On each slice, identify the ligamentum flavum as the hypointense band on the posterolateral wall of the spinal canal, connecting adjacent laminae. |
| 4. Inner boundary | Place the inner point where signal transitions out of the cerebrospinal fluid of the thecal sac. This boundary is defined by an intensity edge (Figure 1D). |
| 5. Outer boundary | Place the outer point at the interface with the lamina. This boundary is NOT marked by an intensity edge, because ligament and cortical bone are both hypointense on T2; the reader must apply a consistent rule. Record the rule used. |
| 6. Orientation | Measure perpendicular to the long axis of the ligament, at the point of maximum extent on that side of that slice. |
| 7. Sampling | Measure bilaterally on every slice spanning the level. Do not measure a single midline point; bilateral all-slice sampling captures asymmetric hypertrophy. |
| 8. Unmeasurable slices | Record as missing rather than substituting a neighbouring value. Do not complete blank cells by estimation. |
| 9. Display | Record the window and level used. Measurement is sensitive to display settings (Supplementary Figure S2B); keep them fixed within and across sessions. |
| 10. Session structure | Record the reading session for every value. If measurement spans more than one session, re-measure a fixed anchor set of slices at the start of each session and record the session offset (Supplementary Figure S2A). |
| 11. Blinding | Measure without access to clinical or radiographic parameters, and for any re-read, without access to prior values, prior slice positions, or algorithmic estimates. |
| 12. Summary | Report the patient-level mean of all readings. Report n readings per patient. |

*Steps 9 and 10 were not applied to the measurements reported in this study and are recommended on the basis of the sensitivity analyses in Supplementary Figure S2.*
