# Supplementary material for: Measurement-level clustering of ligamentum flavum thickness identifies distinct phenotypes associated with lumbar lordosis in adult spinal deformity: a proof-of-concept study
Source: Front Surg. 2026 Oct 14;13:1888647. doi: 10.3389/fsurg.2026.1888647 (PMC13713654; doi:10.3389/fsurg.2026.1888647)
Supplement: Supplementary file 5 [file Table4.docx]

**Supplementary Table S2. Acquisition parameters across the 44 studies.**

| **Parameter** | **Distribution** |
| --- | --- |
| Manufacturer | Siemens 23; GE 15; Hitachi 3; Philips 2; FONAR 1 |
| Field strength | 1.5 T 38; 3.0 T 3; 0.7 T 1; 0.6 T 1; 0.3 T 1 |
| In-plane resolution | 0.281–1.239 mm (22 distinct values); 4.4-fold range |
| Slice thickness | 4.0 mm most common |
| Repetition time | 968–7963 ms |
| Acquisition matrix | 13 distinct matrices |
| Association with mean LF thickness | in-plane resolution r = +0.000, p = 0.999; field strength r = −0.135, p = 0.38 |
| Association with phenotype | resolution p = 0.93; field strength p = 0.21; manufacturer p = 0.96 |

*Per-patient parameters are available from the corresponding author. No examination was excluded for image quality. The single High LF dominant phenotype was imaged at 0.6 T at the coarsest in-plane resolution in the cohort.*
