# Supplementary material for: Measurement-level clustering of ligamentum flavum thickness identifies distinct phenotypes associated with lumbar lordosis in adult spinal deformity: a proof-of-concept study
Source: Front Surg. 2026 Oct 14;13:1888647. doi: 10.3389/fsurg.2026.1888647 (PMC13713654; doi:10.3389/fsurg.2026.1888647)
Supplement: Supplementary file 6 [file Table5.docx]

**Supplementary Table S3. Silhouette sweep for the number of clusters (k = 2 to 5).**

| **k** | **Silhouette** | **Interpretation** |
| --- | --- | --- |
| 2 | 0.598 | Binary (higher score) |
| 3* | 0.573 | Low/Medium/High (selected) |
| 4 | 0.545 | Over-segmented |

**Selected for clinical interpretability. A two-regime solution yielded a marginally higher silhouette value than the three-regime solution retained for the primary analysis; this is reported in Section 3.2 and discussed in Section 4.1.*
