# Supplementary material for: Measurement-level clustering of ligamentum flavum thickness identifies distinct phenotypes associated with lumbar lordosis in adult spinal deformity: a proof-of-concept study
Source: Front Surg. 2026 Oct 14;13:1888647. doi: 10.3389/fsurg.2026.1888647 (PMC13713654; doi:10.3389/fsurg.2026.1888647)
Supplement: Supplementary file 7 [file Table6.docx]

**Supplementary Table S4. Intra-rater reliability by lumbar level.**

| **Level** | **n patients** | **ICC(2,1)** | **Bias, re-read − original (mm)** | **Mean original (mm)** | **Mean re-read (mm)** |
| --- | --- | --- | --- | --- | --- |
| L1-L2 | 15 | 0.222 | +0.09 | 3.03 | 3.12 |
| L2-L3 | 15 | 0.593 | +0.25 | 3.28 | 3.53 |
| L3-L4 | 15 | 0.571 | +0.82 | 3.49 | 4.30 |
| L4-L5 | 15 | 0.543 | +0.40 | 3.37 | 3.77 |
| L5-S1 | 13 | 0.786 | +0.18 | 3.54 | 3.72 |
| Pooled (patient mean) | 15 | 0.632 | +0.36 | 3.34 | 3.70 |

*ICC(2,1): two-way random effects, absolute agreement, single measure. Reliability is lowest at L1-L2 and highest at L5-S1; the pooled figure is dominated by the mid-lumbar levels, which supports level-stratified analysis in future work.*
