# Supplementary material for: Measurement-level clustering of ligamentum flavum thickness identifies distinct phenotypes associated with lumbar lordosis in adult spinal deformity: a proof-of-concept study
Source: Front Surg. 2026 Oct 14;13:1888647. doi: 10.3389/fsurg.2026.1888647 (PMC13713654; doi:10.3389/fsurg.2026.1888647)
Supplement: Supplementary file 8 [file Table7.docx]

**Supplementary Table S5. Distribution of measurement-level thickness regimes across lumbar levels.**

| **Level** | **Measurements (n)** | **Low LF %** | **Medium LF %** | **High LF %** | **Mean ± SD (mm)** |
| --- | --- | --- | --- | --- | --- |
| L1–L2 | 260 | 52.3 | 36.2 | 11.5 | 3.29 |
| L2–L3 | 247 | 50.2 | 40.5 | 9.3 | 3.30 |
| L3–L4 | 252 | 43.7 | 44.4 | 11.9 | 3.46 |
| L4–L5 | 276 | 45.3 | 43.8 | 10.9 | 3.37 |
| L5–S1 | 215 | 57.7 | 33.5 | 8.8 | 3.06 |
| All levels | 1,250 | 49.5 | 39.9 | 10.6 | — |

*Percentages are row-wise within each level. High-regime measurements are distributed across all lumbar levels rather than concentrated at L4-L5.*
