# Supplementary material for: Measurement-level clustering of ligamentum flavum thickness identifies distinct phenotypes associated with lumbar lordosis in adult spinal deformity: a proof-of-concept study
Source: Front Surg. 2026 Oct 14;13:1888647. doi: 10.3389/fsurg.2026.1888647 (PMC13713654; doi:10.3389/fsurg.2026.1888647)
Supplement: Supplementary file 9 [file Table8.docx]

**Supplementary Table S6. PCA variance and domain contributions.**

| **PC** | **Variance** | **Cumulative** | **LF** | **Demo** | **Radio** | **Clin** | **Surg** |
| --- | --- | --- | --- | --- | --- | --- | --- |
| PC1 | 23.9% | 23.9% | 67.0% | 7.6% | 23.5% | 1.0% | 0.9% |
| PC2 | 16.0% | 39.9% | 14.5% | 13.5% | 53.6% | 16.7% | 1.7% |
| PC3 | 10.2% | 50.1% | 10.2% | 50.7% | 20.8% | 11.9% | 6.4% |
| PC4 | 9.2% | 59.3% | 14.0% | 3.7% | 19.6% | 56.4% | 6.3% |

*Domain contributions = % of squared loadings per component. From the original 21-variable analysis; superseded by the reanalysis in Section 3.7.*
