# Supplementary material for: Measurement-level clustering of ligamentum flavum thickness identifies distinct phenotypes associated with lumbar lordosis in adult spinal deformity: a proof-of-concept study
Source: Front Surg. 2026 Oct 14;13:1888647. doi: 10.3389/fsurg.2026.1888647 (PMC13713654; doi:10.3389/fsurg.2026.1888647)
Supplement: Supplementary file 10 [file Table9.docx]

**Supplementary Table S7. Top PCA loadings.**

| **PC** | **Top 3 Loadings** |
| --- | --- |
| PC1 | LF mean (0.41), LF 75% (0.39), LF max (0.34) |
| PC2 | SVA (0.46), TPA (0.44), ODI (0.33) |
| PC3 | Weight (0.46), BMI (0.41), Age (-0.35) |
| PC4 | VAS back (0.57), ODI (0.38), VAS leg (0.31) |

*From the original 21-variable analysis; superseded by the reanalysis in Section 3.7.*
